# Artificial Intelligence Software for Diabetic Eye Screening: Diagnostic Performance and Impact of Stratification

**DOI:** 10.3390/jcm12041408

**Published:** 2023-02-10

**Authors:** Freya Peeters, Stef Rommes, Bart Elen, Nele Gerrits, Ingeborg Stalmans, Julie Jacob, Patrick De Boever

**Affiliations:** 1 Department of Ophthalmology, University Hospitals Leuven, 3000 Leuven, Belgium; 2 Biomedical Sciences Group, Research Group Ophthalmology, Department of Neurosciences, KU Leuven, 3000 Leuven, Belgium; 3 MONA.health, 3060 Bertem, Belgium; 4 Flemish Institute for Technological Research (VITO), 2400 Mol, Belgium; 5 Centre for Environmental Sciences, Hasselt University, Diepenbeek, 3500 Hasselt, Belgium

**Keywords:** diabetes complication, diabetic retinopathy, retina, artificial intelligence, deep learning

## Abstract

Aim: To evaluate the MONA.health artificial intelligence screening software for detecting referable diabetic retinopathy (DR) and diabetic macular edema (DME), including subgroup analysis. Methods: The algorithm’s threshold value was fixed at the 90% sensitivity operating point on the receiver operating curve to perform the disease classification. Diagnostic performance was appraised on a private test set and publicly available datasets. Stratification analysis was executed on the private test set considering age, ethnicity, sex, insulin dependency, year of examination, camera type, image quality, and dilatation status. Results: The software displayed an area under the curve (AUC) of 97.28% for DR and 98.08% for DME on the private test set. The specificity and sensitivity for combined DR and DME predictions were 94.24 and 90.91%, respectively. The AUC ranged from 96.91 to 97.99% on the publicly available datasets for DR. AUC values were above 95% in all subgroups, with lower predictive values found for individuals above the age of 65 (82.51% sensitivity) and Caucasians (84.03% sensitivity). Conclusion: We report good overall performance of the MONA.health screening software for DR and DME. The software performance remains stable with no significant deterioration of the deep learning models in any studied strata.

## 1. Introduction

The number of people with diabetes mellitus (DM) is rapidly increasing, with up to 642 million cases expected by 2040 [1,2]. More than 40% of these diagnosed persons will develop retinopathy. Diabetic retinopathy (DR) and diabetic macular edema (DME) are the main ophthalmological complications of DM, with DR being the leading cause of blindness and visual disability in the working-age population. The risk of such vision loss can be reduced by annual retinal screening and early retinopathy detection to refer cases for follow-up and treatment. The necessary fundus photographs for such screening can be easily obtained non-invasively in an outpatient setting. Implementing a nationwide screening program based on fundus photography resulted in DR no longer being the leading cause of blindness certification in the United Kingdom [3,4,5]. 

However, as long as an ophthalmologist interprets retinal images manually, this screening procedure will always be labor-intensive and expensive, thereby complicating large-scale accessible implementation in many countries. New technologies facilitate the development of care solutions that keep our health system manageable and affordable, especially for diseases of affluence such as DM and associated eye health complications. To realize this ambition, experts in technology and medicine collaborate on solutions to reduce the workload caused by manual grading, a task for which artificial intelligence (AI) is well suited [4,5]. 

AI research in healthcare accelerates with applications achieving human-level performance across various fields of medicine. The use of AI can range from organizational help to surgical applications, with image classification for diagnostic support being one of the main areas of interest [6,7]. IB Neuro™ (Imaging Biometrics, Elm Grove, WI, USA) was the first FDA-approved AI application in 2008 for detecting brain tumors on MRI images. Multiple applications have been approved since then, many in medical imaging domains such as radiology. Some applications go beyond diagnosis and enter therapeutic fields such as radiotherapy [7]. 

Deep learning, a subtype of AI, was introduced not so long ago for the automated analysis and classification of images. In 2016, Gulshan et al. published a landmark paper on a deep learning algorithm with high sensitivity and specificity to classify referable DR [8]. Later papers showed that deep learning algorithms’ diagnostic accuracy is at least comparable to the assessments done by clinicians [9,10,11,12]. Abràmoff and colleagues published their paper on an autonomous AI-based diagnostic system for detecting diabetic retinopathy in 2018 (IDx-DR (Digital Diagnostics, Coralville, IA, USA)). This work led to the first FDA-permitted marketing of an AI-based medical device for automated DR referral [13]. Since then, multiple AI devices have been developed around the world [14].

These developments are exciting, but the clinical community is not yet widely adopting the new tools. Several bottlenecks are at the basis of this hesitation. First, most algorithms are reported by the scientific community and have not been developed into easy-to-use software for primary or secondary care. Second, algorithms mostly report on DR performance, but when considering diabetic eye screening, both DR and DME are relevant. Third, the performance evaluation of the algorithms is done under limited test conditions. Finally, discussions are ongoing at different levels in the healthcare sector about the medico-legal position of AI-based screening and its integration into the patient care path. 

AI accomplishes a specific task on previously curated data, typically from one setting. Ideally, datasets to develop an algorithm are sufficiently diverse to represent the population, with metadata such as age, ethnicity, and sex to allow for performance analysis. In reality, health data lack standardization and contain a bias due to variance in human grading. The actual patient populations are more diverse than those in commonly used datasets [15,16]. Medical data with high-quality labels is challenging to collect, and the General Data Protection Regulation (GDPR) and other privacy-preserving regulations restrict medical data usage. Therefore, most AI models are trained with datasets that have limited heterogeneity. Predictions often do not generalize to different populations or settings. Analyses on subpopulations (e.g., ethnicity) are seldom done, leaving uncertainty that model performance can be reliably extrapolated to new, unseen patient populations [17]. As a result, the performance promised in scientific publications is often not reached in clinical practice, and existing inequalities and biases in healthcare might be exacerbated [17]. Some of these problems can be overcome by executing a prospective clinical trial incorporating pre-chosen metadata and ensuring a relevant distribution amongst specific subpopulations [13]. However, this is a time-consuming and expensive solution, and this approach only allows model evaluation in a limited number of clinical centers. 

International organizations such as the International Diabetes Federation, the International Council of Ophthalmology, the World Council of Optometry, and the International Agency for the Prevention of Blindness support the vast clinical need for widespread and convenient eye health screening tools for persons with diabetes as part of integrated diabetes care [18]. From this perspective, we present an evaluation of a diabetic eye screening software available as a certified medical device for automated DR and DME detection. We report the performance of the deep learning model underlying the software using private and publicly available datasets. Using stratification analyses, we studied the performance in predefined subgroups based on clinically relevant parameters, thereby taking an essential step toward improving the model evaluation process and its robustness during deployment.

## 2. Materials and Methods

### 2.1. MONA.health AI-Based Screening Software

**Figure 1:**
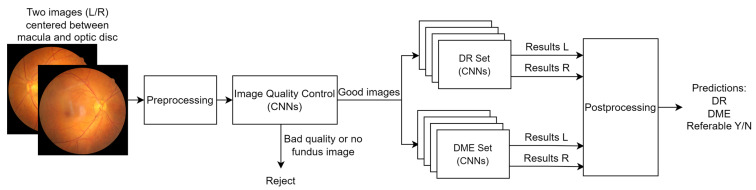
Graphical representation of the MONA.health diabetic eye screening software. A fundus image of each eye is preprocessed, after which an image quality control is executed. The images of sufficient quality are the input to an ensemble of DR models and an ensemble of DME models. L/R: fundus image of the left/right eye.

The MONA.health diabetic eye screening software MONA DR DME (Version 1.0.0; MONA.health, Leuven, Belgium) (https://mona.health/, accessed on 31 January 2023) evaluated in this paper is commercially available as a Class 1 certified medical device under the European Union Medical Device Directive (MDD, Council Directive 93/42/EEC of 14 June 1993 concerning medical devices, OJ No L 169/1 of 7 December 1993). The software needs one fundus image per eye centered between the macula and optic disc for algorithmic processing and reporting three diabetic eye screening results per patient (DR, DME, and a combination of both). The essential processing steps are presented in Figure 1. 

Before presenting the images to the models, they are preprocessed to increase uniformity. This consists mainly of resizing and contrast enhancements, thereby reducing the effects of illumination and fundus pigmentation. Next, the quality of an image is assessed by two models: a model analyzing whether the image is a fundus image or not and a second model evaluating the quality of the image. The second model is trained based on image quality labels according to the EyePACS protocol [19]. An image passing this quality control step is analyzed for referable DR and DME. 

**Figure A1:**
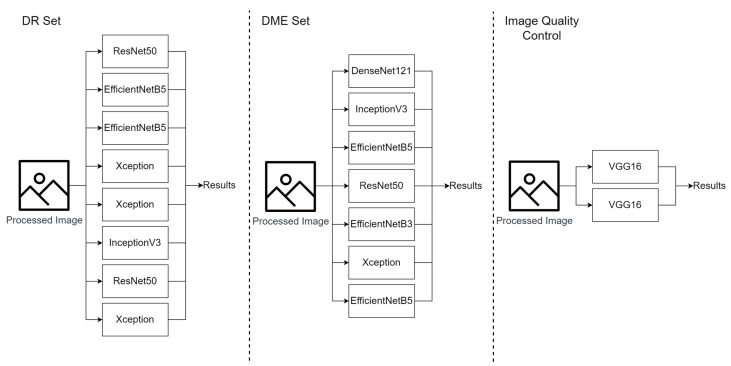
Detailed architecture of the ensembles.

The core of the MONA.health screening software consists of two sets of deep learning models, a DR ensemble and a DME ensemble. Each ensemble is a set of models differing in model architecture and training details such as optimizer, learning rate, and the number of epochs trained. All models used are convolutional neural networks (CNN), with different architectures (ResNet, EfficientNet, Xception, InceptionV3, DenseNet, and VGG). More specifications can be found in Figure A1 [20,21,22,23,24,25]. The results of these individual models are averaged to generate a final output. The resulting output of each ensemble is fundamentally different: an estimation of grade by regression in the case of DR versus a probability of having the disease for DME. Therefore, the models run in parallel instead of having one model that makes all predictions. 

A threshold value was computed for each ensemble to achieve an operating point on the receiver operating curve (ROC) with a sensitivity of 90% for diagnosing referable DR or the presence of DME. These thresholds remained fixed for all subsequent analyses. If the maximal predicted value for at least one eye is higher than these fixed threshold values, the individual is marked for referral for one or both diseases. 

### 2.2. Private Test Set for Algorithm Testing

The fundus images for evaluating the MONA.health diabetic eye screening software originates from the EyePACS telemedicine platform containing patient visits from screening centers in the USA. The characteristics are documented in Table 1. Note that the disease gradings originate from the telemedicine platform without regrading [26]. 

One fundus image per eye, centered between the optic disc and macula, was used for each patient encounter. Relevant metadata, such as age, sex, ethnicity, insulin dependency, and camera type, are available for stratification analysis. The DR grading is consistent with the internationally adopted International Clinical Diabetic Retinopathy (ICDR) severity level [20]. Macular thickening is used in ICDR and Early Treatment Diabetic Retinopathy Study (ETDRS) classification for DME, but cannot be appreciated on standard fundus photographs. Therefore, the presence of hard exudates within one disc diameter of the macula is the surrogate parameter for DME [27,28].

We implemented a filtering procedure to remove images from persons under 18 years, with laser scars, signs of vascular occlusion or cataracts, and images that the image quality models rejected. The image quality models reject poor-quality images for which no interpretation would be possible for a human or an algorithm. Examples of images of sufficient (adequate, good, and excellent) and insufficient quality can be found in Figure A1. The resulting test sets comprised 16,772 patient encounters suitable for DR evaluation (prevalence of referable DR: 48.8%) and 16,833 patient encounters for DME evaluation (prevalence of DME: 11.8%). A total of 16,733 patient encounters were suitable for both evaluations accounting for the large overlap between both datasets.

The MONA.health software performance was evaluated by calculating sensitivity, specificity, positive predictive value (PPV), negative predictive value (NPV), and area under the curve (AUC). These values were calculated for DR, DME, and the combined prediction. The dataset of overlapping patient encounters was used for the combined analysis. Additional analyses were done on ICDR grade 3 and grade 4 retinopathy subgroups since these consist of patients with vision-threatening DR.

### 2.3. Publicly Available Datasets for Algorithm Testing

The second series of evaluations used publicly available datasets containing DR and DME labels at the level of the screened individual. The following datasets were available: the Kaggle DR test set (population: USA; *n* = 5000 patients; multiple cameras) [29], Messidor-2 (population: France; *n* = 874 patients; Topcon NW6) [30,31], and the Messidor-2 Iowa reference (population: France; *n* = 874 patients; Topcon NW6) [32]. The Messidor-2 and Messidor-2 Iowa references use the same image data set but a different grading protocol [32]. 

### 2.4. Stratification Analysis

We performed stratification analyses for patient-based detection of referable DR and DME. The subgroup investigations were done for ethnicity, age, sex, insulin dependency, dilatation status, year of examination, camera type, and image quality. The 95% CIs were calculated via the Percentile Bootstrap Method. The dataset was sampled with replacement for 10,000 repetitions. The size of the sample was always the same size as the data sampled it was from. A sample size calculation was done for the stratified groups based on a pre-specified FDA inferiority hypothesis, namely 75% sensitivity and 78% specificity [13]. A one-sided hypothesis test with binomial distribution was carried out with an overall one-sided 5% Type 1 error, 90% power, and an effect size of 10%. A sample size of 541 evaluated subjects is needed, including 338 subjects with the disease and 203 subjects without the disease to ensure the calculated metrics represent the group. Results for stratified groups with a sample size lower than the computed sample size should be interpreted cautiously since this could be a chance finding. Computations were done in R with the pwr package [33].

## 3. Results

### 3.1. Test Set and Public Datasets

The MONA.health diabetic eye screening software had an excellent performance on the private test set when predicting referable DR and the presence of DME. The area under the curve (AUC) was the primary metric to evaluate the diagnostic prediction, with a patient-based prediction of referable DR of 97.28%. A specificity of 94.62%, a sensitivity of 90.67%, a PPV of 94.14%, and an NPV of 91.40% were recorded for the 90% sensitivity setpoint. The sensitivity was 99.39 and 99.54% when predicting DR grade 3 (severe non-proliferative DR) and grade 4 (proliferative DR), respectively. For DME prediction, the AUC was 98.08% with a specificity of 94.53%, a sensitivity of 90.75%, a PPV of 68.57%, and an NPV of 98.71%. The specificity and sensitivity for the combined DR and DME predictions were 94.24 and 90.91%, respectively. An overview of the performance metrics can be found in Table 2. 

The AUCs obtained on the public datasets were equally high. The values ranged from 96.91 to 97.99% for referable DR. A minimal change in the operating point corresponding to the predefined threshold is noted, as can be observed by inspecting the sensitivity and specificity metrics in Table 2. Similar observations are made for DME on the Messidor-2 dataset. All results are above the proposed minimum requirements set by the FDA in the pre-specified inferiority hypothesis. The evaluation of DME classification could not be reported for the Kaggle test set and Messidor-2 Iowa’s reference because the relevant disease labels are unavailable for these datasets. The referable label in the Iowa reference was based on the assessment of DR and DME and only indicated being referable for either of these diseases. All publicly available datasets were used in their entirety without selection.

### 3.2. Stratification Analysis

**Figure 2:**
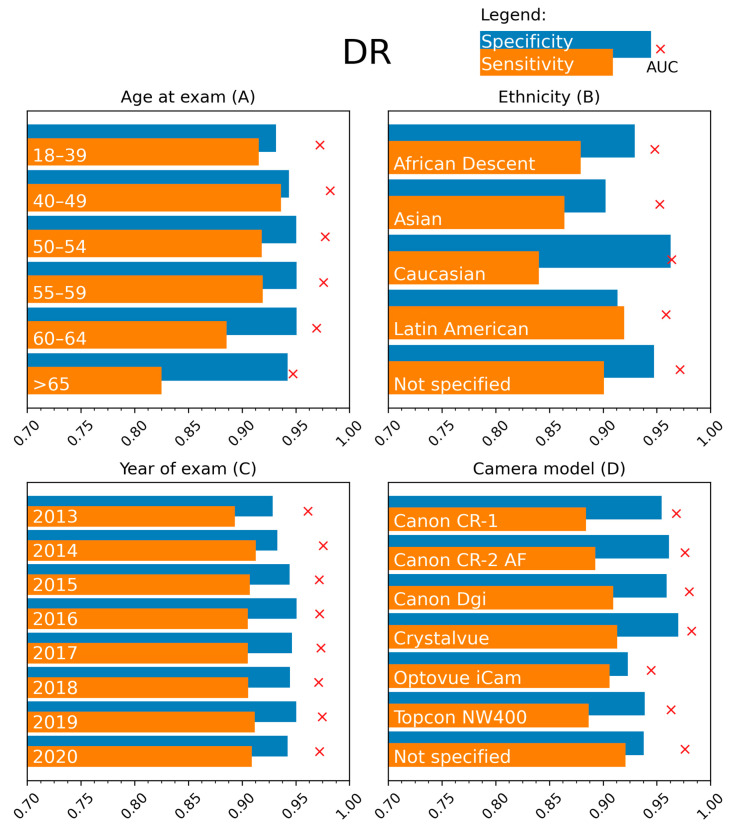
Stratification results: sensitivity (orange), specificity (blue), and AUC (red cross) for detecting referable DR for different subgroups of the test set. The subgroups were created based on presented metadata, namely age at the exam (in years), ethnicity, year of exam, and camera model.

**Figure 3:**
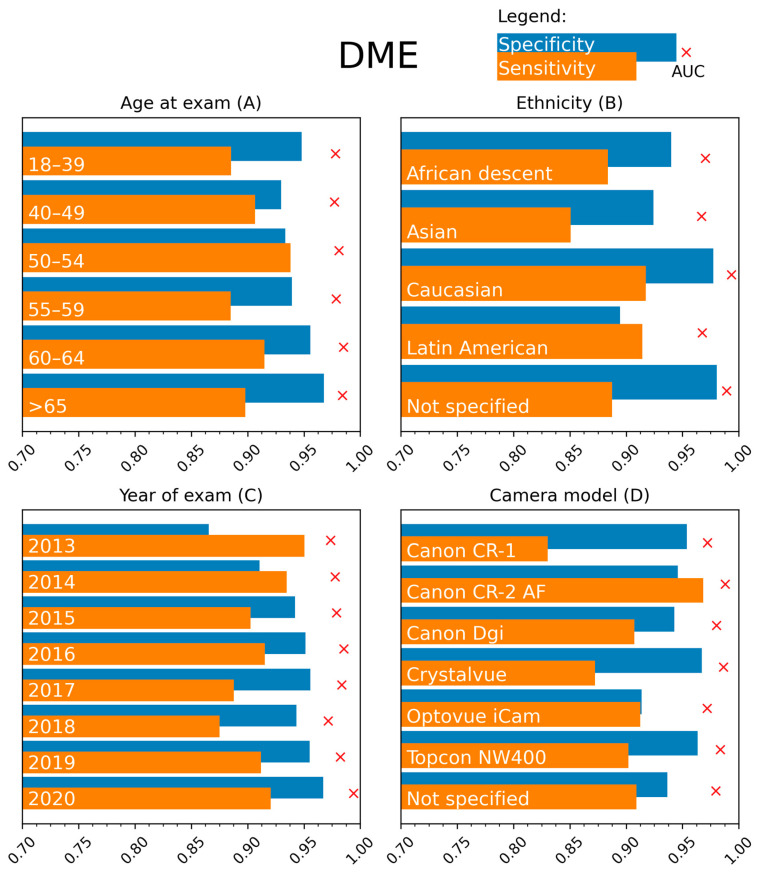
Stratification results: sensitivity (orange), specificity (blue) and AUC (red cross) for DME detection for different subgroups of the test set. The subgroups were created based on presented metadata, namely age at the exam (in years), ethnicity, year of exam, and camera model.

We report the sensitivity and specificity for detecting referable DR (Figure 2) and DME (Figure 3) when dividing the private test set into subgroups according to attributes relevant to the persons with diabetes and the eye screening procedure. The results are for the model with the fixed threshold computed for the 90% sensitivity setpoint. Detailed numerical values of the analysis are in Appendix A (Table A1, Table A2, Table A3, Table A4, Table A5, Table A6, Table A7 and Table A8).

A high sensitivity (exceeding 90% on average) for detecting referable DR is obtained for most age groups, with only a decreased sensitivity of 82.51% (95% confidence intervals can be found in Appendix A) in the 65+ age group. Specificity remained high at 94.24% in this age group. No differences between the age groups are encountered for DME detection. 

DR referral in the groups defined based on ethnicity (Figure 2B) had a high AUC of 96.38% observed in the Caucasian group, with lower values in the Asian (95.26%) and African (94.80%) subpopulations. However, sensitivity values are lower in the Caucasian (84.03%) and higher in the Latin American (91.95%) populations. The AUC was high for all subgroups (range 96.67–99.34%) for DME referral (Figure 3B). Decreased sensitivity and specificity are noted in the Asian population and lower specificity in the Latin American population. 

The diabetic eye screening software showed excellent overall performance, without any relevant differences when the dataset was divided according to the sex or insulin dependency status of the patients. A difference in sensitivity/specificity division can be perceived at the 90% sensitivity operating point in the latter group for DR. Considering DME, a lower specificity of 91.72% was noted in the insulin-dependent group compared to 95.74% in the non-insulin-dependent group. We refer the reader to Appendix A for detailed reports on this analysis. 

Stratifying the data according to the year of examination showed good performances for DR referral, with a slight decrease in sensitivity to 89.32% for the oldest images (Figure 2C). A high sensitivity but lower specificity is observed in this group for DME (Figure 3C). The dilatation status during fundus photographing did not affect the model performance (Appendix A). 

The AUCs were comparable between the different fundus cameras, with values between 96.30 and 98.22%, except for the Optovue iCam 100 (Visionix, Pont-de-l’-Arche, France) (94.46%). High sensitivity is observed for DME using the Canon CR-2 camera (Canon, Tokyo, Japan) (96.84%), while the values for the other cameras ranged from 83.04 to 91.25% (Figure 3D). Sensitivity for DME is lower for images obtained on the Canon CR-1 (Canon, Tokyo, Japan) (83.04%) camera. 

## 4. Discussion

We report a systematic retrospective evaluation of the MONA.health diabetic eye screening software that analyzes fundus images using artificial intelligence and summarizes DR and DME classification outputs as a single result to assess the patient referral status. Our investigations were performed on a large, multi-center, private test set from a US-based screening network and publicly available datasets regularly used to benchmark diabetic eye detection algorithms. The private test set reported 90.91% sensitivity and 94.24% specificity for referring a person because of DR or DME. These values are higher than the pre-specified superiority endpoints of 85% sensitivity and 82.5% specificity proposed in the work of Abràmoff and coworkers [13]. It is relevant to say that the latter values are for a prospective study while we performed a retrospective study. Nevertheless, our performances are comparable to previously published work [13,34,35,36,37,38,39,40,41]. Our study adds value to the research field by reporting the results of data stratification to study differences in model performance in subpopulations. Such an analysis is essential to assess the usability of the software in clinical practice, thereby providing a starting point for better insights into potential hurdles when incorporating AI-based decision support software in clinical practice. 

All DR grades beyond mild DR are considered referable and justify a physical examination by an ophthalmologist. However, the higher the retinopathy grade, the higher the risk of vision loss and the more urgent the need for referral. Therefore, high sensitivities are even more critical for detecting severe non-proliferative DR and proliferative DR. Sensitivities of 99.39 and 99.54% were obtained for these cases of vision-threatening DR, indicating that the vast majority of cases will be accurately referred by the software. A substantial difference in PPV, the probability that subjects with a positive screening test truly have the disease, is noted when considering the diagnosis of DME (68.75%) compared to DR (94.14%). This difference is likely attributed to the lower disease prevalence of DME (11.76%) in the test set. 

The performance was analyzed on the publicly available Kaggle, Messidor-2, and Messidor-2 Iowa reference datasets. The algorithm has a robust performance, with only slight decreases in AUC and sensitivity for DR on the publicly available Kaggle test set. This observation may be attributed to the fact that the Kaggle test set only contains images dating before 2015 [29]. We observed a comparable decrease in sensitivity in our test set for older images (Figure 2C). For the Messidor-2 dataset, AUC values are comparable to those reported on the test set for the regular and Iowa reference. However, a decrease in specificity and an increase in sensitivity are noted for DR. This rebalance between sensitivity and specificity indicates that the chosen threshold is suboptimal for this specific dataset. These findings are consistent with those of Gulshan et al. [8]. A possible explanation for this shift in operating point is the homogeneity of the dataset (one camera type and only patients from France with a less diverse ethnic mix) [30,31]. However, the chosen threshold might still result in a performance closer to the 90% sensitivity operating point in a more variable real-life setting than shown on the Messidor-2 dataset. This hypothesis is supported by the analysis results on the more extensive test set. A decreased sensitivity for DME is observed on the Messidor-2 data compared to our own test set. A shift in operating point is the most likely explanation for this observation. This effect is larger in the Iowa reference labeling. This might be attributed to a difference in labeling between these two references. For the same images, the patient level prevalence is 21.7% in the Iowa labeling [32] compared to 30.9% in the standard labeling (calculated based on [8]).

The performance evaluations of AI algorithms detecting DR and DME can yield good results, but guaranteeing high model performance for all relevant subpopulations is still a significant challenge. We performed an extensive stratification analysis in the current study to investigate possible differences in performance. This evaluation has not been reported to our knowledge. 

The algorithm’s performance for DR and DME classification was stable in the different age categories up to 65 years. Beyond the patient age of 65 years, a decrease in sensitivity for DR detection to 82.51% was recorded. Acquiring high-quality fundus images can be more challenging in the elderly due to patient-related factors such as corneal changes, vitreous floaters, and cataract formation. However, the lower sensitivity in DR detection cannot be solely attributed to this factor since no remarkable differences were noted in the stratification analysis based on image quality. No alternative explanations could be found based on the performed stratifications. 

The MONA.health software is registered as diabetic eye screening software in Europe. Of note, ethnicity distribution is different between the European and USA-based populations of the private test set. An ethnicity stratification was performed to aid the software performance evaluation. The biggest relevant deviations were found for sensitivity in the Caucasian subgroup (84.03%) and specificity in the Latin American subgroup (91.33%). A detailed analysis of the positive cases in this Caucasian group was done, showing that for Caucasians, 33.3% of the referable cases are based on the presence of retinal hemorrhages with/without micro-aneurysms without any other lesion types (such as cotton wool spots, hard exudates, IRMA, venous beading, new vessels, fibrous proliferation, preretinal, or vitreous hemorrhage). By comparison, this is only the case in 23.5% of all non-Caucasian cases and 22.2% of Latin American cases (the largest subgroup amongst positive cases). We assume DR detection is more difficult in the Caucasian population due to a lower prevalence of other signs besides hemorrhages. Our medical retina experts’ analysis of all Caucasian false negatives revealed that dust spots and shadows had been mislabeled as hemorrhages. Previous research showed that artifacts might be an important reason for intra- and interobserver variability and mislabeling [15]. Nevertheless, the achieved performances remain above the non-inferiority hypothesis [13]. 

The prevalence of referable DR is higher in the Latin American population than in the Caucasian population [42]. Increased prevalence may be associated with a higher likelihood of more severe disease, which is more easily detected [43,44]. This might contribute to the observed differences. Furthermore, 30% of patients are of “unspecified” ethnic origin, making many images unavailable for the stratification analysis. A drop in specificity for the Latin American subgroup is observed. Considering that the AUC remains high in this group, this observation may indicate that there is a more optimal threshold for this subgroup. The high disease prevalence might reinforce this effect in this subgroup. Sensitivity and specificity metrics for the Asian and African subgroups should be interpreted cautiously. The sample size of these two groups was under the minimal sample size of 541, making it hard to draw any meaningful conclusion. 

Multiple parameters were explored to stratify the analysis for disease severity. Due to the low quality of specific labels such as HbA1c values and years since diagnosis (missing data, impossible values), these parameters were not kept for analysis. Therefore, insulin dependency was selected as a surrogate parameter for disease severity. This stratification showed a difference in sensitivity/specificity division for DR (93.94/88.55% vs. 87.81/96.22%), meaning that the ideal operating point for 90% sensitivity differs between the two groups. In real life, only one threshold can be used, and a mix between insulin-dependent and independent patients is expected, balancing the differences between both groups. 

Considering the year of examination, intuitively, one would expect a lower performance when analyzing older images since image quality, resolution, and ease of use have increased over the years due to technological improvements. This statement appears to hold for DR. However, for DME, increased sensitivity and decreased specificity are seen for the older images. At the same time, the AUC remained high, indicating that older images might also benefit from a different threshold. No notable discrepancies between results were recorded when considering camera type. Regarding DME, a lower sensitivity was observed for the Canon CR-1 camera (Canon, Tokyo, Japan). 

This study comes with strengths and limitations. We report the performance of the MONA.health software that uses one fundus image of the left eye and one of the right eye to generate a report about the patient’s referral status for DR and DME. One fundus image per eye results in higher patient comfort and lower operational costs, making the software easy to use. This software was developed explicitly for diabetic eye screening, and its operational settings balance sensitivity, specificity, and cost-effectiveness [45]. The referral threshold was computed and subsequently fixed for subsequent usage in the software [45]. An additional study strength is the evaluation of the software using a sizable private test set and publicly available datasets. Furthermore, the stratification analysis investigated the diagnostic performance of such an AI-based algorithm for the first time. Overall, we report stable high-performance results using widely used metrics such as AUC, sensitivity, and specificity. We highlight the importance of stratification from a research and clinical perspective by illustrating potential hurdles to overcome before implementing AI in daily practice. The stratification illustrates that comparisons based on AUC can be deceiving since most strata have a very high AUC, but the resulting performances for a predefined threshold may shift. In a production setting, one cannot tailor this threshold to the specific needs of the context since this would require a new and elaborate validation study to prove effectiveness [45]. 

The most critical limitation of stratification is that results depend on the initial label’s quality both for the ground truth of the diagnosis and for the metadata. Our research team obtained the private test set from the well-established EyePACS telemedicine platform. The EyePACS protocols for collecting fundus images and diabetic eye screening are reliable. However, the protocols were initially not designed to organize metadata for later use in a stratification analysis to assess AI-based image analysis. We noted several problems regarding this quality during our study, such as impossible numerical values and missing data. A more robust higher quality dataset would be necessary to further improve research on this subject. Nonetheless, patient consent and privacy issues limit obtaining such a dataset, and a post hoc curation of an existing dataset is extremely difficult. A second limitation is the difference between prevalence in the dataset (48.8%) and real-life prevalence, of which reports vary but are considerably lower [46,47,48,49,50,51]. We considered correcting for this difference in our study, but it was decided not to rebalance the dataset to maintain a sufficient number of images. Finally, prospective studies and post-market clinical evaluations are needed to evaluate MONA-health software performance further and support our conclusions. Such studies are currently underway and indexed as clinical trials NCT05260281 and NCT05391659. 

## 5. Conclusions

We present a detailed evaluation of the MONA.health AI screening software for detecting referable DR and DME using a single fundus image per eye. Performance analysis shows good overall results. An extensive stratification analysis considered patient characteristics and parameters related to eye screening. We observed variability between the results of the subgroups, but overall performance remained stable with no significant deterioration of the deep learning model in any of the studied strata. We advocate that reporting stratification performances is essential when envisioning a DR screening algorithm in clinical practice, but such results are typically not reported. Our research highlights the importance of high-quality data, thereby forming a basis for the improvement of future research in medical AI by bringing to attention some of its current shortcomings. 

## Figures and Tables

**Table 1:** An overview of the study population. The category other consists of invalid values, errors, impossible values, etc.

Characteristics	Dataset DR	Dataset DME
Unique patient visits	16,772	16,833
Diagnosis	Non-referable DR (*n* = 8581)	DME (*n* = 1979)
	Referable DR (*n* = 8191)	No DME (*n* = 14,854)
Age at examination	18–39 (*n* = 1575)	18–39 (*n* = 1572)
	40–49 (*n* = 3532)	40–49 (*n* = 3539)
	50–54 (*n* = 2858)	50–54 (*n* = 2870)
	55–59 (*n* = 3309)	55–59 (*n* = 3323)
	60–64 (*n* = 3062)	60–64 (*n* = 3080)
	>65 (*n* = 2436)	>65 (*n* = 2449)
Sex	Female (*n* = 8431)	Female (*n* = 8460)
	Male (*n* = 7410)	Male (*n* = 7439)
	Other (*n* = 931)	Other (*n* = 934)
Ethnicity	African (*n* = 506)	African (*n* = 512)
	Asian (*n* = 461)	Asian (*n* = 463)
	Caucasian (*n* = 3040)	Caucasian (*n* = 3048)
	Latin American (*n* = 6394)	Latin American (*n* = 6418)
	Not specified (*n* = 5769)	Not specified (*n* = 5790)
	Other (*n* = 602)	Other (*n* = 602)
Insulin dependency	Yes (*n* = 5567)	Yes (*n* = 5574)
	No (*n* = 11,058)	No (*n* = 11,113)
	Other (*n* = 147)	Other (*n* = 146)
Dilatation	Yes (*n* = 7411)	Yes (*n* = 7858)
	No (*n* = 8725)	No (*n* = 8975)
	Other (*n* = 636)	Other (*n* = 0)
Camera type	Canon CR-1 (*n* = 1194)	Canon CR-1 (*n* = 1196)
	Canon CR-2 AF (*n* = 1007)	Canon CR-2 AF (*n* = 1009)
	Canon DGi (*n* = 2468)	Canon DGi (*n* = 2478)
	Crystalvue (*n* = 470)	Crystalvue (*n* = 473)
	Optovue iCam (*n* = 1462)	Optovue iCam (*n* = 1468)
	Topcon NW400 (*n* = 3107)	Topcon NW400 (*n* = 3125)
	Not specified (*n* = 6374)	Not specified (*n* = 6399)
	Other (*n* = 690)	Other (*n* = 685)
Image quality grading [18]	Adequate (*n* = 5810)	Adequate (*n* = 5829)
	Good (*n* = 6767)	Good (*n* = 6787)
	Excellent (*n* = 4195)	Excellent (*n* = 4217)
Year of examination	2013 (*n* = 699)	2013 (*n* = 667)
	2014 (*n* = 1422)	2014 (*n* = 1432)
	2015 (*n* = 2549)	2015 (*n* = 2561)
	2016 (*n* = 2818)	2016 (*n* = 2830)
	2017 (*n* = 3169)	2017 (*n* = 3173)
	2018 (*n* = 2627)	2018 (*n* = 2638)
	2019 (*n* = 2791)	2019 (*n* = 2802)
	2020 (*n* = 727)	2020 (*n* = 730)

**Table 2:** Performance evaluation results (given in %) for the test set and the public datasets. Results are for the selected 90% sensitivity operating point. AUC: area under the curve; Spec.: specificity; Sens.: sensitivity; Acc.: accuracy; N/A: not applicable; NR: not relevant. DR grade 3 refers to severe non-proliferative DR and grade 4 to proliferative DR.

Dataset	Disease	AUC % (95% CI)	Spec. % (95% CI)	Sens. % (95% CI)	Acc. % (95% CI)	Sens. DR Grade 3 % (95% CI)	Sens. DR Grade 4 % (95% CI)
Private test set	DR (*n* = 16,772)	97.28 (97.50–97.52)	94.62 (94.12–95.08)	90.67 (90.03–91.31)	92.69 (92.28–93.09)	99.39 (98.88–99.80)	99.54 (98.83–100.00)
	DME (*n* = 16,833)	98.08 (97.85–98.30)	94.46 (94.08–94.83)	90.75 (89.46–92.01)	94.02 (93.66–94.39)	NR	NR
	DR + DME (*n* = 16,733)	N/A	94.24 (93.75–94.73)	90.91 (90.28–91.54)	92.62 (92.22–93.02)	99.39 (98.87–99.80)	99.54 (98.80–100.00)
Kaggle test set (*n* = 26,788)	DR	96.91 (96.63–97.18)	95.16 (94.87–95.44)	88.45 (87.63–89.28)	93.68 (93.39–93.97)	98.81 (97.92–99.56)	99.74 (99.32–100)
	DME	N/A	N/A	N/A	N/A	NR	NR
Messidor-2 (*n* = 870)	DR	97.99 (97.08–98.76)	92.86 (90.73–94.83)	93.66 (90.62–96.46)	93.10 (91.38–94.71)	100 (100–100)	100 (100–100)
	DME	98.98 (98.31–99.50)	99.35 (98.70–99.87)	74.04 (65.26–82.29)	96.34 (94.94–97.47)	NR	NR
	DR + DME	N/A	93.00 (90.92–94.97)	93.70 (90.68–96.48)	93.22 (91.49–94.83)	100 (100–100)	100 (100–100)
Messidor-2 Iowa’s reference (*n* = 874)	DR + DME	N/A	84.06 (81.30–86.72)	97.89 (95.65–99.51)	87.07 (84.90–89.24)	NR	NR

**Figure A2:**
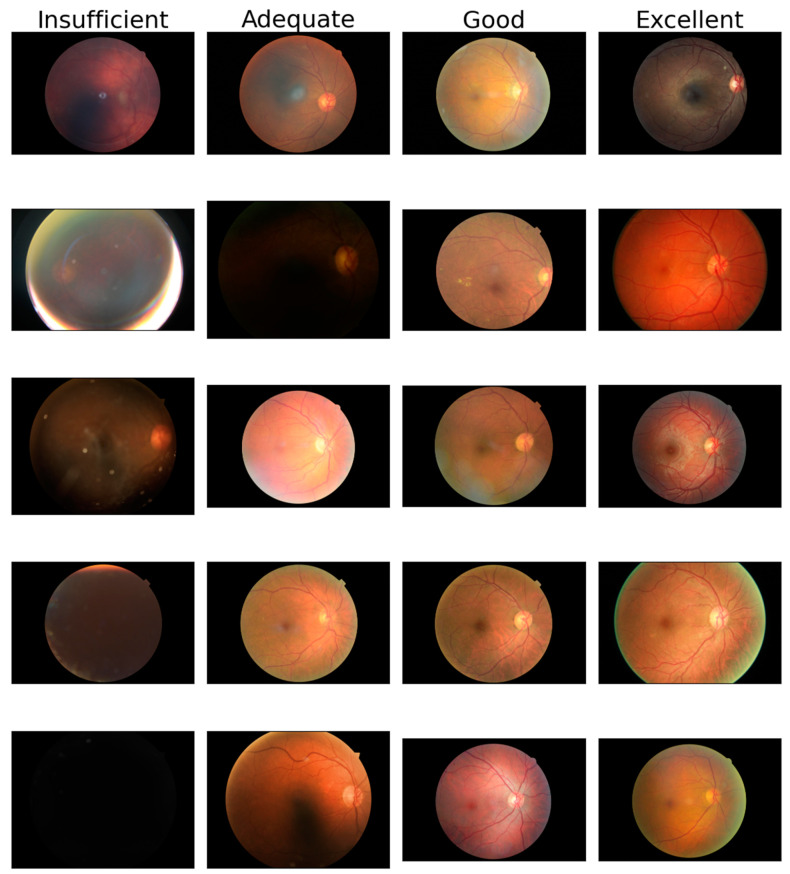
Examples of images of insufficient, adequate, good, and excellent quality.

## Data Availability

Publicly available datasets were analyzed in this study. These data can be found here: https://www.adcis.net/en/third-party/messidor2/ (accessed 11 April 2022); https://medicine.uiowa.edu/eye/abramoff (accessed 11 April 2022); https://www.kaggle.com/competitions/diabetic-retinopathy-detection/data (accessed 11 April 2022).

## Appendix A

**Table A1:** Stratification results for detecting referable retinopathy and diabetic macular edema on the patient level per age group.

Disease	Group	Sample Size	Sample Size %	Prevalence %	AUC % (95% CI)	Sensitivity % (95% CI)	Specificity % (95% CI)
DR	18–39 years	1575	9.39	48.89	97.23 (96.48–97.93)	91.56 (89.58–93.48)	93.17 (91.35–94.87)
	40–49 years	3532	21.06	56.43	98.20 (97.83–98.56)	93.63 (92.54–94.69)	94.35 (93.16–95.46)
	50–54 years	2858	17.04	54.76	97.73 (97.21–98.23)	91.82 (90.44–93.17)	95.05 (93.84–96.18)
	55–59 years	3309	19.73	48.35	97.58 (97.07–98.06)	91.94 (90.56–93.28)	95.08 (94.06–96.09)
	60–64 years	3062	18.26	43.66	96.92 (96.30–97.49)	88.56 (86.86–90.27)	95.07 (94.03–96.08)
	≥65 years	2436	14.52	38.01	94.73 (93.78–95.59)	82.51 (80.00–84.89)	94.24 (93.06–95.38)
DME	18–39 years	1572	9.34	9.41	97.79 (96.91–98.54)	88.51 (83.03–93.43)	94.80 (93.63–95.92)
	40–49 years	3539	21.02	13.90	97.70 (97.16–98.20)	90.65 (87.99–93.13)	92.98 (92.04–93.87)
	50–54 years	2870	17.04	14.67	98.10 (97.56–98.57)	93.82 (91.40–96.01)	93.34 (92.36–94.30)
	55–59 years	3323	19.74	12.82	97.85 (97.29–98.34)	88.50 (85.40–91.38)	93.92 (93.04–94.77)
	60–64 years	3080	18.30	9.94	98.51 (97.99–98.95)	91.50 (88.24–94.48)	95.57 (94.78–96.32)
	≥65 years	2449	14.55	7.59	98.40 (97.63–99.03)	89.78 (85.19–93.99)	96.77 (96.04–97.48)

**Table A2:** Stratification results for detecting referable retinopathy and diabetic macular edema on the patient level for ethnicity.

Disease	Group	Sample Size	Sample Size %	Prevalence %	AUC % (95% CI)	Sensitivity % (95% CI)	Specificity % (95% CI)
DR	African Descent	506	3.02	71.94	94.80 (92.60–96.68)	87.91 (94.45–91.12)	92.96 (88.49–96.88)
	Asian	461	2.75	73.32	95.26 (93.23–97.03)	86.39 (82.69–90.00)	90.24 (84.62–95.28)
	Caucasian	3040	18.13	18.95	96.38 (95.39–97.27)	84.03 (80.97–86.94)	96.27 (95.50–97.00)
	Latin American	6394	38.12	84.13	95.86 (95.09–96.57)	91.95 (91.22–92.67)	91.33 (89.52–93.03)
	Not specified	5769	34.40	17.80	97.16 (96.57–97.71)	90.07 (88.18–91.89)	94.73 (94.08–95.35)
DME	African Descent	512	3.04	21.88	97.01 (95.36–98.38)	88.39 (81.98–94.07)	94.00 (91.56–96.17)
	Asian	463	2.75	14.47	96.67 (94.70–98.29)	85.08 (76.12–93.15)	92.42 (89.75–94.95)
	Caucasian	3048	18.11	4.36	99.34 (99.04–99.60)	91.73 (86.82–96.06)	97.74 (97.18–98.27)
	Latin American	6418	38.13	20.02	96.74 (96.26–97.17)	91.44 (89.86–92.98)	89.46 (88.61–90.28)
	Not specified	5790	34.40	4.46	98.91 (98.43–99.31)	88.76 (84.72–92.43)	98.05 (97.67–98.41)

**Table A3:** Stratification results for the detection of referable retinopathy and diabetic macular edema on the patient level for sex.

Disease	Group	Sample Size	Sample Size %	Prevalence %	AUC % (95% CI)	Sensitivity % (95% CI)	Specificity % (95% CI)
DR	Female	8431	50.27	46.98	97.05 (96.70–97.39)	89.78 (88.82–90.72)	94.77 (94.10–95.41)
	Male	7410	44.18	53.52	97.49 (97.14–97.81)	91.40 (90.52–92.26)	94.60 (93.83–95.33)
DME	Female	8460	50.26	10.87	98.19 (97.84–95.81)	90.65 (88.71–92.48)	95.23 (94.74–95.70)
	Male	7439	44.19	13.28	97.84 (97.48–98.17)	90.89 (89.08–92.61)	93.35 (92.73–93.94)

**Table A4:** Stratification results for the detection of referable retinopathy and diabetic macular edema on the patient level for insulin dependency.

Disease	Group	Sample Size	Sample Size %	Prevalence %	AUC % (95% CI)	Sensitivity % (95% CI)	Specificity % (95% CI)
DR	Insulin-dependent	5567	33.19	67.83	96.70 (96.21–97.17)	93.94 (93.15–94.69)	88.55 (87.08–89.98)
	Not insulin-dependent	11,058	65.93	39.25	97.11 (96.79–97.42)	87.81 (86.83–88.77)	96.22 (95.76–96.67)
DME	Insulin-dependent	5574	33.11	17.19	97.01 (96.53–97.45)	90.50 (88.56–92.32)	91.72 (90.92–92.51)
	Not insulin-dependent	11,113	66.02	9.04	98.57 (98.29–98.82)	90.95 (89.15–92.74)	95.74 (95.35–96.12)

**Table A5:** Stratification results for the detection of referable retinopathy and diabetic macular edema on the patient level for year of exam.

Disease	Group	Sample Size	Sample Size %	Prevalence %	AUC % (95% CI)	Sensitivity % (95% CI)	Specificity % (95% CI)
DR	2013	669	3.99	89.54	96.14 (93.91–97.96)	89.32 (86.79–91.74)	92.86 (86.05–98.46)
	2014	1422	8.48	64.49	97.56 (96.77–98.27)	91.28 (89.35–93.08)	93.27 (91.04–95.36)
	2015	2549	15.20	47.82	97.18 (96.57–97.77)	90.73 (89.04–92.33)	94.44 (93.20–95.65)
	2016	2818	16.80	46.56	97.20 (96.60–97.78)	90.55 (88.94–92.16)	95.09 (93.96–96.18)
	2017	3169	18.89	43.01	97.33 (96.78–97.83)	90.54 (88.97–92.07)	94.63 (93.56–95.63)
	2018	2627	15.66	48.38	97.12 (96.48–97.70)	90.56 (88.93–92.14)	94.47 (93.20–95.65)
	2019	2791	16.64	44.25	97.44 (96.86–97.97)	91.17 (89.58–92.74)	95.05 (93.94–96.12)
	2020	727	4.33	37.83	97.22 (95.91–98.30)	90.91 (87.37–94.16)	94.25 (92.01–96.30)
DME	2013	667	3.96	24.29	97.36 (95.97–98.46)	95.06 (91.33–98.12)	86.54 (83.50–89.43)
	2014	1432	8.51	14.94	97.76 (96.98–98.43)	93.46 (90.00–96.52)	91.05 (89.40–92.65)
	2015	2561	15.21	11.64	97.90 (97.25–98.47)	90.27 (86.83–93.42)	94.22 (93.24–95.14)
	2016	2830	16.81	12.08	98.54 (98.03–98.97)	91.52 (88.45–94.30)	95.14 (94.28–95.97)
	2017	3173	18.85	10.12	98.36 (97.95–98.76)	88.79 (85.29–92.13)	95.58 (94.79–96.32)
	2018	2638	15.67	11.22	97.16 (96.35–97.88)	87.50 (83.56–91.22)	94.32 (93.36–95.25)
	2019	2802	16.65	10.10	98.22 (97.55–98.79)	91.17 (87.72–94.38)	95.51 (94.71–96.30)
	2020	730	4.34	8.63	99.40 (98.94–99.76)	92.06 (84.75–98.28)	96.70 (95.29–97.94)

**Table A6:** Stratification results for the detection of referable retinopathy and diabetic macular edema on the patient level per camera model.

Disease	Group	Sample Size	Sample Size %	Prevalence %	AUC % (95% CI)	Sensitivity % (95% CI)	Specificity % (95% CI)
DR	Canon CR-1	1194	7.12	37.60	96.82 (95.72–97.82)	88.42 (85.49–91.28)	95.44 (93.91–96.87)
	Canon CR-2	1007	6.00	53.72	97.60 (96.65–98.43)	89.28 (86.57–91.88)	96.14 (94.31–97.84)
	Canon Dgi	2468	14.72	43.40	98.02 (97.52–98.49)	90.94 (89.23–92.66)	95.92 (94.83–96.93)
	Crystalvue	470	2.80	29.36	98.22 (96.61–99.44)	91.30 (86.33–95.65)	96.99 (94.94–98.75)
	Optovue iCam	1462	8.72	86.66	94.46 (92.43–96.24)	90.61 (88.94–92.17)	92.31 (88.44–95.71)
	Topcon NW400	3107	18.52	45.41	96.30 (95.62–96.92)	88.66 (87.01–90.26)	93.87 (92.70–94.96)
	Not specified	6374	38.00	48.10	97.60 (91.11–93.03)	92.07 (91.11–93.03)	93.77 (92.92–94.58)
DME	Canon CR-1	1196	7.11	9.36	97.21 (95.75–98.42)	83.04 (75.68–89.82)	95.39 (94.08–96.60)
	Canon CR-2	1009	5.99	15.66	98.80 (98.23–99.28)	96.84 (93.92–99.35)	94.60 (93.01–96.05)
	Canon Dgi	2478	14.72	11.74	98.02 (97.40–98.58)	90.72 (87.31–93.94)	94.28 (93.31–95.25)
	Crystalvue	473	2.81	9.94	98.63 (97.82–98.83)	87.23 (76.74–95.92)	96.71 (94.87–98.32)
	Optovue iCam	1468	8.72	21.80	97.17 (96.27–97.97)	91.25 (88.04–94.26)	91.38 (89.70–92.96)
	Topcon NW400	3125	18.56	6.21	98.36 (97.82–98.83)	90.21 (85.79–94.24)	96.35 (95.67–97.01)
	Not specified	6399	38.01	12.74	97.94 (97.53–98.31)	90.92 (88.93–92.81)	93.70 (93.07–94.32)

**Table A7:** Stratification results for the detection of referable retinopathy and diabetic macular edema on the patient level for image quality.

Disease	Group	Sample Size	Sample Size %	Prevalence %	AUC % (95% CI)	Sensitivity % (95% CI)	Specificity % (95% CI)
DR	Adequate	5810	34.64	49.07	97.04 (96.61–97.44)	91.06 (89.98–92.08)	93.82 (92.94–94.68)
	Good	6767	40.35	49.12	97.19 (96.81–97.55)	90.28 (89.27–91.25)	94.89 (94.14–95.60)
	Excellent	4195	25.01	48.06	97.79 (97.39–98.17)	90.77 (89.50–92.00)	95.27 (94.35–96.13)
DME	Adequate	5829	34.63	11.75	97.22 (96.71–97.71)	87.88 (85.42–90.29)	93.66 (92.98–94.32)
	Good	6787	40.32	12.30	98.60 (98.33–98.85)	92.22 (90.36–94.00)	95.03 (94.48–95.56)
	Excellent	4217	25.05	10.88	98.28 (97.79–98.72)	92.38 (89.91–94.71)	94.65 (93.91–95.37)

**Table A8:** Stratification results for the detection of referable retinopathy and diabetic macula edema on the patient level for dilation status.

Disease	Group	Sample Size	Sample Size %	Prevalence %	AUC % (95% CI)	Sensitivity % (95% CI)	Specificity % (95% CI)
DR	Dilated	7820	46.63	53.96	96.88 (96.51–97.24)	91.07 (90.19–91.90)	93.14 (92.30–93.94)
	Undilated	8952	53.37	44.36	97.67 (97.38–97.95)	90.25 (89.30–91.18)	95.68 (95.12–96.25)
DME	Dilated	7858	46.68	12.66	98.20 (97.89–98.49)	92.26 (90.55–93.86)	94.40 (93.85–94.94)
	Undilated	8975	53.32	10.96	97.97 (97.62–98.29)	89.23 (87.26–91.12)	94.51 (94.00– 94.99)
